# Cell wall anisotropy plays a key role in *Zea mays* stomatal complex movement: the possible role of the cell wall matrix

**DOI:** 10.1007/s11103-023-01393-x

**Published:** 2023-12-18

**Authors:** K. Gkolemis, E. Giannoutsou, I-D. S. Adamakis, B. Galatis, P. Apostolakos

**Affiliations:** Section of Botany, Department of Biology, School of Sciences, National and Kapodistrian University of Athens, Athens, Greece

**Keywords:** *Zea mays*, Stomatal complexes, Homogalacturonans, Cell wall expansion, Anisotropy, Cell wall matrix

## Abstract

The opening of the stomatal pore in *Zea mays* is accomplished by the lateral displacement of the central canals of the dumbbell-shaped guard cells (GCs) towards their adjacent deflating subsidiary cells that retreat locally. During this process, the central canals swell, and their cell wall thickenings become thinner. The mechanical forces driving the outward displacement of the central canal are applied by the asymmetrically swollen bulbous ends of the GCs via the rigid terminal cell wall thickenings of the central canal and the polar ventral cell wall (VW) ends. During stomatal pore closure, the shrinking bulbous GC ends no longer exert the mechanical forces on the central canals, allowing them to be pushed back inwards, towards their initial position, by the now swelling subsidiary cells. During this process, the cell walls of the central canal thicken. Examination of immunolabeled specimens revealed that important cell wall matrix materials are differentially distributed across the walls of *Z. mays* stomatal complexes. The cell walls of the bulbous ends and of the central canal of the GCs, as well as the cell walls of the subsidiary cells were shown to be rich in methylesterified homogalacturonans (HGs) and hemicelluloses. Demethylesterified HGs were, in turn, mainly located at the terminal cell wall thickenings of the central canal, at the polar ends of the VW, at the lateral walls of the GCs and at the periclinal cell walls of the central canal. During stomatal function, a spatiotemporal change on the distribution of some of the cell wall matrix materials is observed. The participation of the above cell wall matrix polysaccharides in the well-orchestrated response of the cell wall during the reversible movements of the stomatal complexes is discussed.

**Supplementary Information:**

The online version contains supplementary material available at 10.1007/s11103-023-01393-x.

## Key message

The involvement of various cell wall matrix materials in the cell wall anisotropy presented in guard and subsidiary cells during *Zea mays* stomatal complex movement.

**Supplementary Information:**

The online version contains supplementary material available at 10.1007/s11103-023-01393-x.

## Introduction

The guard cells (GCs) of stomatal complexes are one of the few plant cell types that can reversibly change their shape and volume during their functional cycles. The GC cell walls must therefore possess unique structural properties to withstand and facilitate the mechanics of stomatal movements (Wu and Sharpe 1979; Rui et al. 2018). In vascular plants, two major types of GCs can be distinguished: kidney-shaped GCs and dumbbell-shaped GCs. Most vascular plants display kidney-shaped GCs, while the dumbbell-shaped GCs appear in the Poaceae family. In the kidney-shaped GCs, the change in GC shape is the outcome of a strictly controlled interplay of the protoplast, which undergoes reversible turgor pressure changes, with the surrounding cell walls, resulting in the opening and the closure of the stomatal pore. A major role in this process is played by the periclinal GC cell walls which are reinforced with unique radial cellulose microfibril systems (Ziegenspeck 1939; Galatis and Mitrakos 1980) deposited under the guidance of respectively organized microtubule arrays (Galatis and Mitrakos 1980; Galatis and Apostolakos 2004; Marom et al. 2017; Woolfenden et al. 2017; Bidhendi and Geitmann 2018). More recent works on kidney-shaped GCs have additionally shown that particular matrix cell wall materials are involved in the opening and closure of the stomatal pore, by locally altering the mechanical properties of the cell walls (reviews by Marom et al. 2017; Woolfenden et al. 2017; Rui et al. 2018; Apostolakos et al. 2021). Most interestingly, Carter et al. (2017) have demonstrated a possible relationship between the increased stiffness of the polar regions of kidney-shaped *Arabidopsis* stomata and the deposition of demethylesterified homogalacturonans (HGs) in these areas.

The dumbbell-shaped GCs of the Poaceae are comprised of two enlarged bulbous ends bridged by an elongated central region, the central canal (Esau 1965). The latter is narrow and displays intensely thickened periclinal cell walls, while the bulbous ends display relatively thinner cell walls. Local cell wall thickenings emerging from the central canal, described as terminal thickenings of the central canals, are directed towards the junction sites of the lateral anticlinal cell walls of the bulbous ends with their periclinal ones (Galatis 1980). Cellulose microfibrils in the cell wall thickenings of the central canal as well as at its terminal thickenings tend to be aligned along the stomatal axis. The periclinal cell walls of the bulbous ends display cellulose microfibril systems radiating out from the margins of the central canal towards the GC ends (Ziegenspeck 1939; Galatis 1980). Once again, these cellulose microfibril arrangements were deposited under the guidance of respective radial cortical microtubule arrays (Galatis 1980; Galatis and Apostolakos 2004).

In contrast to the kidney-shaped stomata, the mechanics of the opening and closing of the stomatal pore in dumbbell-shaped ones appear to be more complex and are not yet completely understood. It has, however, been well established that the radial cellulose microfibril systems in the periclinal walls allow for the swelling of the bulbous ends when the turgor pressure rises, which leads to the opening of the stomatal pore. When the turgor pressure drops, the bulbous ends reacquire their initial shape and the stomatal pore closes. Moreover, Franks and Farquhar (2007) and Giannoutsou et al. (2020) have shown that during the opening of the stomatal pore in *Triticum aestivum* and *Z. mays*, the central canals of the GCs are laterally displaced “into the subsidiary cells’’, which are conveniently deformed to accommodate their displacement (see also Chen et al. 2017). To put it briefly, the central canals of the GCs “invade into the subsidiary cells”, because forces generated by the swollen bulbous ends cause the central canals to move outwards (Apostolakos et al. 2021). As it was initially observed by Aylor et al. (1973) and restated by Apostolakos et al. (2021), the swelling of the bulbous ends seems to happen asymmetrically, rather than symmetrically. Up to this day, Aylor et al. (1973) have been the first and only researchers to clearly state that while the GCs of closed grass stomata are indeed shaped like dumbbells, the same is not true for the GCs of open grass stomata. In their own words: “the outline of a telephone receiver could better describe the shape of a guard cell in an open *Zea mays* stoma” (Aylor et al. 1973).

Regarding the GC wall matrix composition in dumbbell-shaped stomata, Jones et al. (2005) have noted that the GC walls of *Z. mays* are rich in arabinose, xylose and phenolic pectin esters. The distribution of HGs displaying different degrees of methylesterification has, moreover, been studied in developing and mature stomatal complexes of *Z. mays* (Giannoutsou et al. 2016, 2020). It has been suggested that methylesterified HGs contribute to the expansion of the cell walls during GC morphogenesis and stomatal pore formation, while pectins with a high degree of demethylesterification allow certain GC cell wall regions to withstand the mechanical forces exerted during stomatal function. Callose distribution has also been studied in developing and mature stomatal complexes of *Z. mays*, revealing that, in mature stomatal complexes, callose enriches the cell wall thickenings of the central canal, the terminal cell wall thickenings of the central canal and the polar ventral wall (VW) ends (Giannoutsou et al. 2020; Apostolakos et al. 2021). Callose deposition in these regions probably locally increases the stiffness of the cell walls, making them rigid enough to secure the lateral displacement of the central canal “into the subsidiary cells” during stomatal pore opening.

Apart from data presented so far, little information has been published on the importance of the cell wall matrix during the movements of dumbbell-shaped stomata. However, the possible role of several cell wall matrix components in cell wall mechanical behavior has been extensively discussed in recent scientific literature, even if mostly in regard to cells and tissues which undergo irreversible expansion. Xyloglucans, for instance, which are recognized by the LM15 monoclonal antibody (Marcus et al. 2008), are thought to contribute to cell wall extensibility (Whitney et al. 1999; Park and Cosgrove 2012a,b; Rui and Anderson 2016), through their interaction with cellulose microfibrils. (1,3;1,4)-β-d-glucans (mixed linkage glucans-MLGs), which like xyloglucans are hemicelluloses, appear to be involved in cell wall expansion through the promotion of cell wall elasticity and malleability (Carpita et al. 2001; Buckeridge et al. 2004; Fincher 2009; Harris and Fincher 2009; Chang et al. 2021). Their topological distribution can be assessed by immunolabeling with the BG-1 monoclonal mouse antibody (Biosupplies, Australia Ltd), which recognizes linear (1,3;1,4)-β-oligosaccharide fragments. HGs, on the other hand, are pectic macromolecules whose effect on the mechanical properties of the cell wall mainly depends on their degree of methylesterification. Highly methylesterified HGs tend to increase cell wall extensibility (Verhertbruggen and Knox 2007; Wolf and Greiner 2012; Bidhendi and Geitmann 2016), whereas HGs that have undergone blockwise demethylesterification are generally associated with increased cell wall rigidity due to their ability to become cross-linked through Ca^2+^ ionic bridges (Willats et al. 2001; Cosgrove 2005; Verhertbruggen and Knox 2007; Palin and Geitmann 2012; Bidhendi and Geitmann 2018). Highly methylesterified HGs are recognized by the LM20 (Verhertbruggen et al. 2009) and JIM7 antibodies (Knox et al. 1990). JIM5 on the other hand, functions as a general molecular probe against HGs with a low degree of methylesterification (Knox et al. 1990), while the 2F4 antibody is specific for demethylesterified HGs cross-linked with Ca^2+^ (Liners et al. 1989).

This work attempts to investigate the role of cell wall mechanics in the accomplishment of reversible stomatal movement in dumbbell-shaped grass stomata. Therefore, the following parameters were studied in the stomatal complexes of *Z. mays*: (a) The changes in the shape of the cells comprising the stomatal complex that occur during the opening and closing of the stomatal pore, and (b) the distribution of several different cell wall matrix materials, namely HGs, xyloglucans, and MLGs, in the cell walls of open and closed stomatal complexes.

## Materials and methods

### Plant material

This study was carried out in leaves of young seedlings of *Z. mays* L. var. Aris. Seedlings were grown in wrapped sheets of filter paper soaked with distilled water, which were placed in glass containers for 6–8 days, in darkness, at 25 ± 1 °C. These seedlings were used for the preparation of hand-made paradermal leaf sections or for embedding in LRW resin. Stomatal opening was induced in humid conditions created in covered glass containers. Free hand-made leaf sections were preferentially made in the morning hours when the stomatal aperture was obviously enhanced. Such a differentiation of stomatal response in relation to a circadian rhythm is in accordance with previously published data on the subject (Hubbard and Webb 2015; Resco de Dios 2017). When stomatal closure was required, the steps of the method favoring stomatal opening were omitted.

### Measurement of selected morphological parameters in closed and open stomatal complexes

All measurements performed to quantify changes in the shape and the dimensions of the component cells of stomatal complexes during the stomatal movements were carried out in images of 100 closed and 100 open stomata seen at median paradermal optical section. Closed stomata were sampled from 12 individual plants while open stomata were sampled from 11 individual plants. Hand-made paradermal sections were taken from both first and second leaves of each *Z. mays* seedling. All sections used for measurements were not fixed, to avoid interference with stomatal aperture. Instead, they remained in distilled water during observation and photography under a Differential Interference Contrast (DIC) optical system. Analysis and statistic evaluation of the acquired measurements was done with Sigma Plot Ver. 12.0 (Systat Software, San Jose, CA).

### Immunolocalization of HGs and xyloglucans

Immunolocalization of HG epitopes: LM20 (Verhertbruggen et al. 2009; Frey et al. 2023); JIM7 (Knox et al. 1990); JIM5 (Knox et al. 1990; Frey et al. 2023); 2F4 (Liners et al. 1989) and xyloglucans (LM15) (Marcus et al. 2008) was carried out in hand-made paradermal sections of fresh leaf tissue, which were initially fixed in 4% (w/v) paraformaldehyde (PFA) in PEM buffer (50 mm PIPES [piperazine-N,N′-bis(2-ethanesulphonic acid)], 5 mm EGTA, 5 mm MgSO4, pH 6.8) for 45 min. Following fixation, the sections were washed three times with PEM for 15 min and treated with 2% (w/v) cellulase (Onozuka R-10 [Yakult Honsha Co. L, Tokyo, Japan]) in PEM, pH 5.6, for 60 min. After being washed again with PEM, the sections were extracted with 0.5% (v/v) Triton X-100 and 2% (v/v) dimethylsulphoxide (DMSO) in Phosphate Buffer Saline (PBS) for 20 min. Subsequently, they were washed thrice with PBS and then incubated overnight with the respective primary antibody. All primary antibodies apart from 2F4 were diluted 1:40 in PBS. 2F4 was diluted 1:40 in T/Ca/S buffer (Tris–HCl 20 mM pH 8.2, CaCl2 0.5 mM, NaCl 150 mM), for which reason three additional washes with T/Ca/S buffer were performed before incubation with the antibody. On the following day, the sections were rinsed with PBS or, in the case of the 2F4 primary antibody, with T/Ca/S buffer and incubated with a secondary antibody for 3 h at 37 °C. For all primary antibodies apart from 2F4 a FITC–conjugated anti-rat IgG (Sigma) diluted 1:40 in PBS was used. 2F4 was instead recognized by a FITC–conjugated anti-mouse IgG (Sigma) diluted 1:40 in T/Ca/S buffer. After washing with the appropriate buffer, the specimens were finally mounted with a mixture of glycerol/PBS (2:1 v/v) containing 0.5% (w/v) p-phenylenediamine (anti-fade medium).

### Immunolocalization of mixed linkage glucans (MLGs)

Immunolocalization of MLGs in semithin sections was performed on small leaf pieces fixed in 2% (w/v) PFA and 0.1% (v/v) glutaraldehyde in PEM at 4 °C for 1.5 h. The specimens were washed in the same buffer and dehydrated in a graded ethanol series (10–90%) diluted in distilled water before being transferred in absolute ethanol. Dehydration in absolute ethanol was repeated thrice for 30 min at a time. During dehydration the specimens were kept at 0 °C and were additionally post-fixed with 0.25% (w/v) osmium tetroxide added to the 30% ethanol step for 2 h. After dehydration, the material was infiltrated with LR White (LRW) (Sigma) acrylic resin diluted in ethanol, in 10% steps to 100% (1 h in each step), at 4 °C and with pure resin overnight. The samples were then embedded in gelatin capsules filled with LRW resin and polymerized at 60 °C for 48 h.

Semithin sections (approximately 2–3 μm thick) of material embedded in LRW resin were transferred to glass slides and blocked with 5% (w/v) BSA in PBS for 5 h. After washing with PBS, BG-1 antibody (Biosupplies, Australia Ltd) diluted 1:40 in PBS containing 2% (w/v) BSA was applied overnight. Following rinsing with PBS and blocking again with 2% (w/v) BSA in PBS, the sections were incubated for 3 h, at 37 °C in FITC anti-mouse IgG (Sigma) diluted 1:40 in PBS containing 2% (w/v) BSA. After rinsing with PBS, the sections were mounted using an anti-fade medium.

### Callose localization

In living stomatal complexes, callose was localized in paradermal hand-made leaf sections stained with 0.05% (w/v) aniline blue (Sigma, C.I. 42,725) in 0.07 M K_2_HPO_4_ buffer, pH 8.5 (O’Brien and McCully 1981). Sections remained in aniline blue solution during observation at the epifluorescent microscope.

Callose immunolocalization in semithin sections of material embedded in LRW resin was achieved by the protocol previously described for the immunolabeling of MLGs. Callose was targeted by an anti-callose mouse antibody, which was in turn recognized by a FITC anti-mouse IgG. Both the primary and the secondary antibody were diluted 1:40 in PBS containing 2% (w/v) BSA.

### CBM-mediated immunolabeling of crystalline cellulose

The immunolabeling of crystalline cellulose in living stomatal complexes required the succession of three molecular probes. In place of a primary antibody, a His-tagged CBM3 carbohydrate binding module was used (Pfaff et al. 2022; Zhang et al. 2018). The His-tag was recognized by a mouse anti-His-tag antibody, which was in turn targeted by a FITC-conjugated anti-mouse IgG (Sigma). All probes were diluted 1:40 in PBS. Steps preceding or following incubation with each of the probes were similar to those described for HG and xyloglucan immunolocalization with the exception of the enzyme treatment where a solution of 2% (w/v) macerozyme (Macerozyme R-10 [Yakult Honsha Co. Ltd, Tokyo, Japan] in PEM was used instead of a cellulase solution.

### Transmission electron microscopy (TEM)

In preparation for observation by transmission electron microscopy (TEM), small pieces of *Z. mays* leaves were fixed in glutaraldehyde, post-fixed in osmium tetroxide, dehydrated in an acetone series and embedded in either Durcupan ACM (Fluka) or Spurr’s resin (Serva, Heidelberg, Germany). Ultrathin sections were stained with uranyl acetate and lead citrate.

### Observation and photography

The specimens were examined with a Zeiss Axioplan microscope equipped with a UV source, a Differential Interference Contrast (DIC) optical system, and a Zeiss Axiocam MRc5 digital camera. Two filter sets were used for the specimens’ observation: a filter set provided with exciter solid glass filter 365 nm and barrier long-wave pass band filter 420 nm, and another set provided with exciter pass band filter 450–490 nm and barrier pass band filter 515–565 nm. Series of paradermal optical sections were studied, to ascertain the distribution of the examined cell wall materials along the whole surface of the cell walls, while at least three independent experiments were conducted for the localization of each cell wall epitope in *Z. mays* stomatal complexes. More than 20 sections taken from 3 to 6 plants were examined in every experiment. All samples were checked for UV autofluorescence using the above filters. Thin sections of the embedded specimens were examined with a Philips 300 TEM. Furthermore, some specimens were examined with a confocal laser scanning microscope (CLSM; BC 43 Benchtop Confocal Microscope, Andor Technology, Belfast, Ireland; https://andor.oxinst.com/products/bc43-benchtop-confocal-microscope).

### Quantification of fluorescence

Quantification of fluorescence in the periclinal cell walls of the central canal and of the subsidiary cells was performed in ImageJ/FIJI after the immunolocalization of pectin-epitopes with the LM20, JIM7, JIM5 and 2F4 antibodies. Areas of interest were traced by using the ImageJ Freehand Selection tool and mean grey value per pixel was measured and then corrected by background subtraction. Mean background gray value was determined by sampling and averaging the mean gray values of 3–5 background areas that, while part of the leaf tissue, were distinct from the area of interest and did not display immunofluorescence. Corrected mean gray value was subsequently converted to corrected integrated density per μm^2^. Pixels to μm^2^ scaling was achieved by use of the Straight-Line tool and the Set Scale function. Each measurement was repeated in at least 3 comparable images and a mean value ± standard error (SE) for the corrected integrated density per μm^2^ was then extracted (Zhu et al. 2015).

## Results

### Guard cell wall terminology

**Fig. 1:**
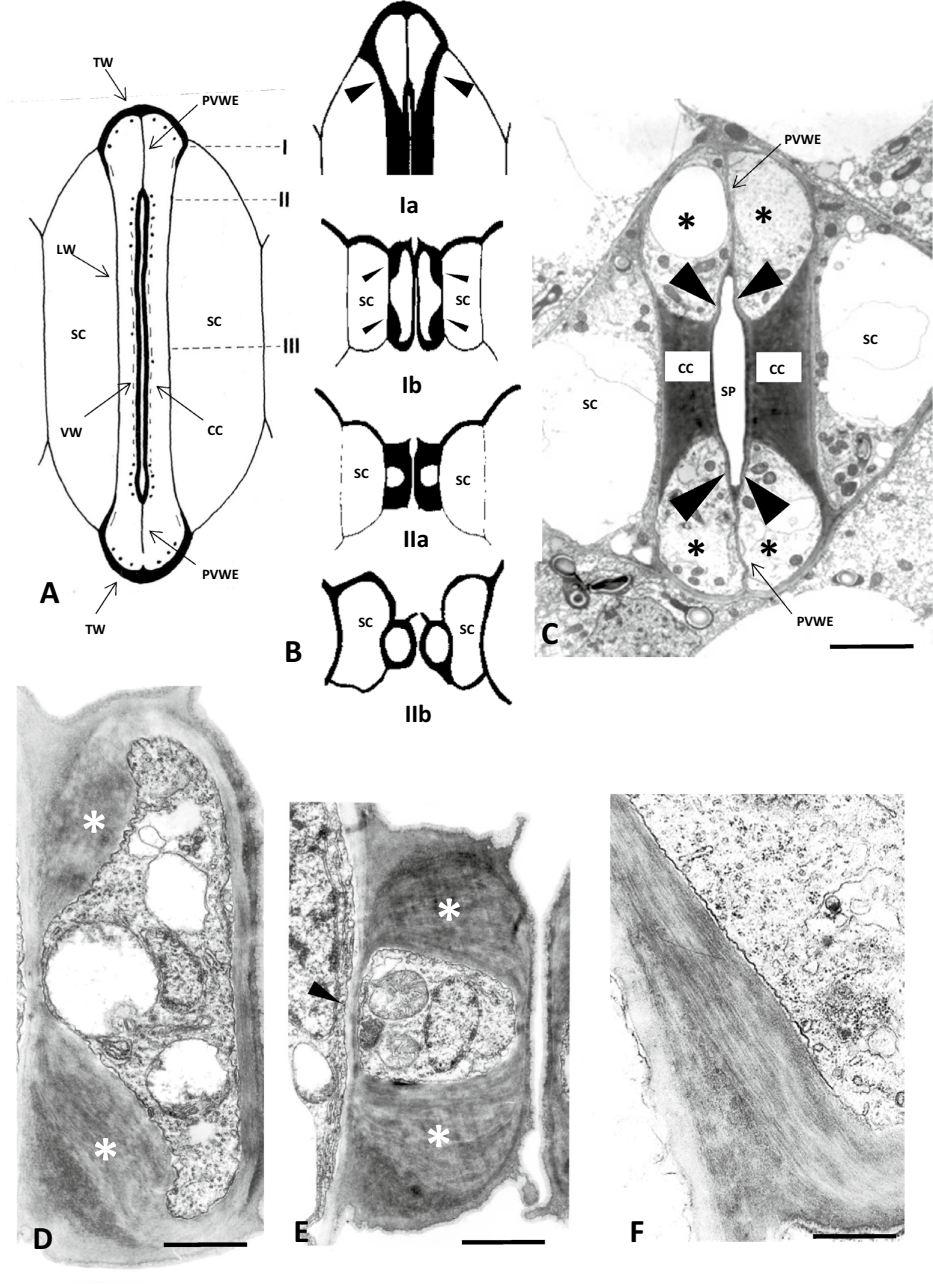
**A** Diagrammatic representation of a closed *Z. mays* stomatal complex in median paradermal view (from Galatis 1980). The dots represent microtubules. *CC* Central Canal, *PVWE* Polar Ventral Cell Wall End, *LW* Lateral Cell Wall, *TW* Transverse Cell Wall, *SC* Subsidiary Cell, *VW* Ventral Cell Wall. **B** (Ia) Diagrammatic representation of an external paradermal section of area I of Figure A. The arrowheads point to terminal cell wall thickenings of the central canal. (Ib) Diagrammatic representation of a transverse section of a stomatal complex. The section corresponds to the plane II of Figure A. The arrowheads point to the terminal cell wall thickenings of the central canal. (IIa) Diagrammatic representation of the stomatal complex in transverse section corresponding to the middle of the central canal (plane III in A). The intense cell wall thickenings of the central canal are shown. *SC* Subsidiary Cell. (IIb) Diagrammatic representation of an opening stomatal complex. The section corresponds to the middle of the central canal. Compare to IIa. *SC* Subsidiary Cell. **C** Paradermal section of *Z. mays* stomatal complex as depicted in TEM. The asterisks mark the bulbous ends of the GCs. The arrowheads point to the parts of the ventral cell wall between the central canal (CC) and the polar ends of the ventral cell wall (PVWE). *SC* subsidiary cell. Scale bar: 5 μm. **D** Transverse GC section as seen in TEM. The section corresponds to plane II of A. The asterisk mark the terminal cell wall thickenings of the central canal. Scale bar: 2 μm. **E** GC as depicted in TEM in transverse section passing through the middle of the central canal. Note the intense cell wall thickenings (asterisks). Scale bar: 2 μm. **F** Terminal cell wall thickening of the central canal as depicted in TEM in longitudinal section. Note the cellulose microfibrils orientation. Scale bar: 1 μm

The adjacent anticlinal GC walls, between which the stomatal pore is created, represent the ventral cell walls (VW; Fig. 1A). The VW portions extending from the stomatal pore towards the stomatal ends are defined as the polar VW ends (PVWE; Fig. 1A). The anticlinal cell wall of the GCs which is opposite to the VW is called lateral (LW), while those that are vertical to the VW are called transverse cell walls (TW) (Fig. 1A). The cell walls of the stomatal complex running parallel to the leaf surface are described as the periclinal cell walls.

### Morphological changes of the stomatal complex cells during the opening and closure of the stomatal pore

The structure of the GCs at the closed stomata of *Z. mays* has extensively been studied in TEM by Srivastava and Sing (1972) and Galatis (1980). In Fig. 1C, a paradermal *Z. mays* stomatal complex section is shown, where the central canal with its thickened cell walls as well as the bulbous ends can clearly be observed. In Fig. 1E, a transverse section of the central canal is depicted, where the intense cell wall thickenings of the periclinal cell walls can be observed (see also Fig. 1BIIa). In Fig. 1D, a transverse section of a GC is shown at the region of the terminal cell wall thickenings that emerge from the central canal (see also Fig. 1BIb). In Fig. 1BIa, a diagrammatic representation of the terminal cell wall thickenings of the central canal in paradermal section is shown and in Fig. 1F, one of these thickenings as seen in TEM.

**Fig. 2:**
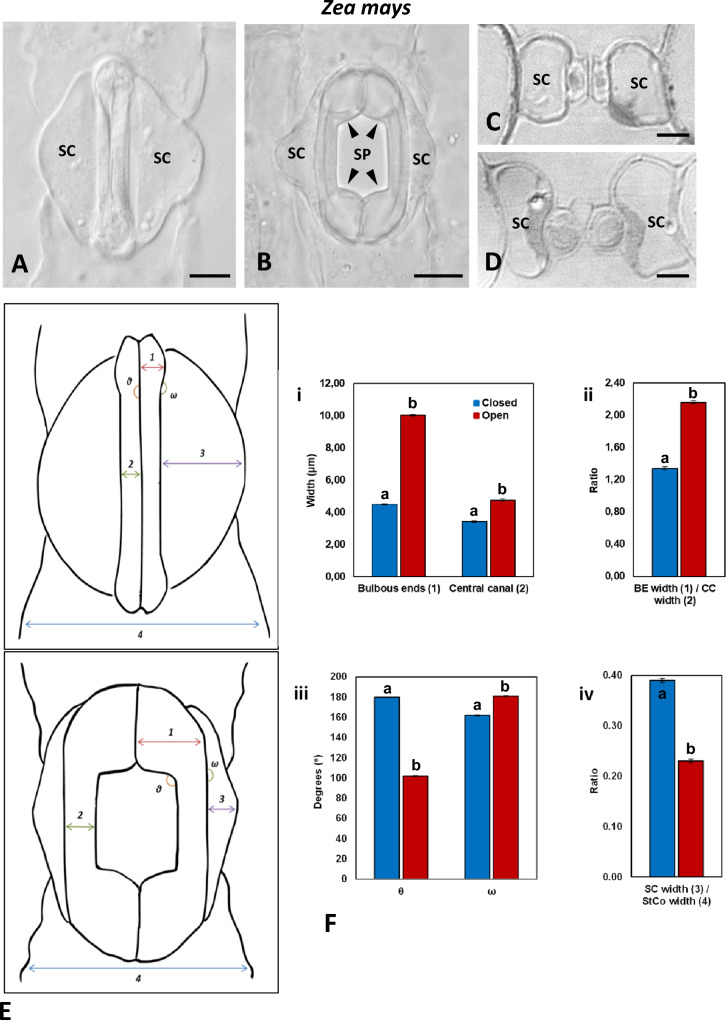
**A**, **B**: Closed (**A**) and open (**B**) stomatal complex as seen in DIC in paradermal view. The arrowheads mark parts of the VW that elongate during the opening of the stomatal pore. *SC* Subsidiary Cell, *SP* Stomatal Pore. Scale bars: 10 μm. **C**, **D**: Transverse sections of a closed stomatal complex (**C**) and an opening stomatal complex (**D**) as seen in DIC. The sections pass through a median plane of the central canal. *SC* Subsidiary Cell. Scale bars: 5 μm. **E** Diagrammatic representation of a closed and an open stomatal complex. **1** width of bulbous end (BE width), **2** width of central canal (CC width), **3** width of subsidiary cell (SC width), **4** overall width of stomatal complex (StCo width), **θ°** angle between the ventral wall of the central canal and the ventral wall of the bulbous end, **ω°** angle between the lateral wall of the central canal and the lateral cell wall of the bulbous end. **F** Graphic comparison of the mean values of measured morphological parameters in closed (blue columns) and open stomata (red columns): **i** BE width and CC width, **ii** ratio of BE width to CC width, **iii** angles θ° and ω°, **iv** ratio of SC width to StCo width. Error bars on each column represent standard error. Numbers included in parentheses in the legends of the horizontal axis refer to each parameter’s numbering in **E**. Different letters state a statistically significant difference (P ≤ 0.001)

According to measurements carried out in the present study, in closed stomata, the width of the central canal (N.2 in Fig. 2E) is 3.43 ± 0.04 μm (n = 100, mean ± SE), while the width of the bulbous end (N.1 in Fig. 2E) is 4.49 ± 0.03 μm (n = 100). Furthermore, the height of the bulbous ends, i.e., the dimension that is vertical to the leaf surface, is larger than that of the central canal (Fig. 1BIb; cf. Figure 1BIIa), while their external periclinal cell wall lies on a higher plane than that of the central canal (Suppl. Fig. 1A). In the bulbous ends, the polar VW ends display large gaps (Srivastava and Singh 1972; Galatis 1980), enabling the GC pair to function as a unit. Finally, in the closed stomata, the subsidiary cells acquire a triangular shape in paradermal view (Fig. 2A); their maximum width (N.3 in Fig. 2E) is 12.16 ± 0.18 μm (n = 100). In these stomata, the fraction of the subsidiary cell’s width to the whole width of the stomatal complex (N.4 in Fig. 2E) is 0.39 ± 0.003 (Fig. 2F iv). Our data showed that, in closed stomata, the angle formed between the VW and the polar VW ends, shown as θ° in Fig. 2E, is 180° (n = 100; Fig. 2F iii), while the angle formed between the lateral cell wall of the central canal and the lateral cell wall of the bulbous ends, shown as ω° in Fig. 2E, is 162° (n = 100; Fig. 2F iii).

**Fig. 3:**
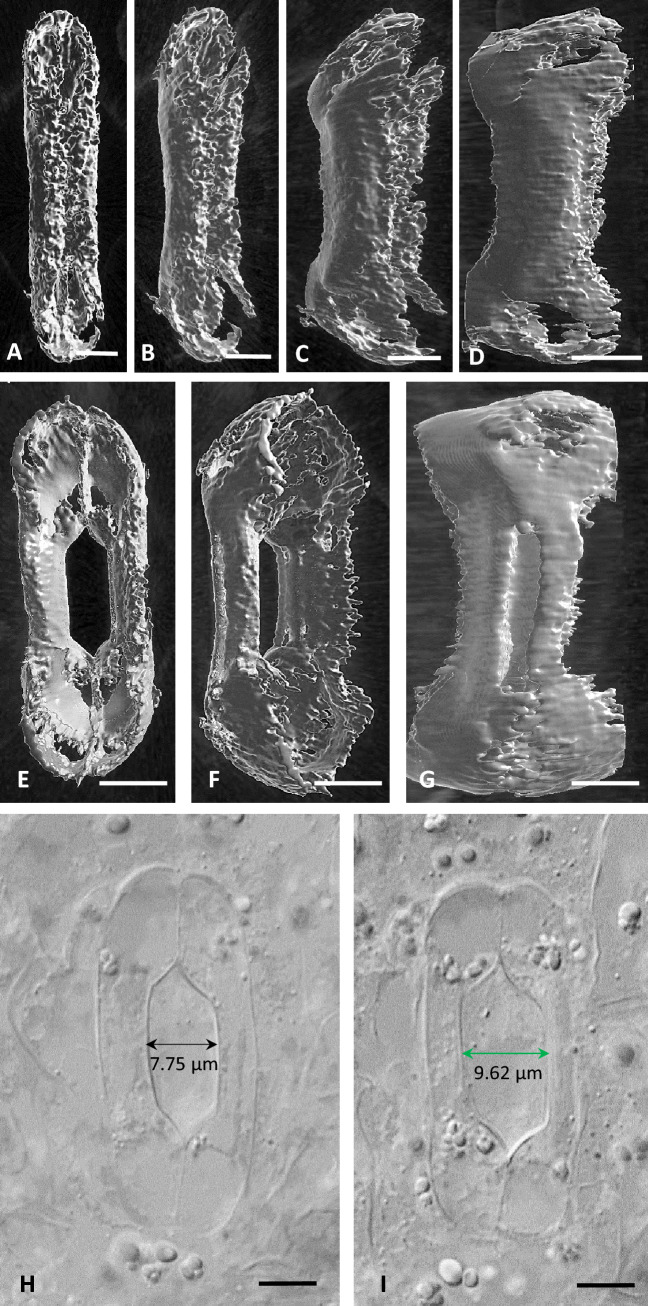
**A**–**D** Successive captures of 3D reconstruction of a closed stoma using pictures taken by BC-43 confocal microscope. **E**–**G**: Successive captures of 3D reconstruction of an open stoma using pictures taken by BC-43 confocal microscope. **H**, **I**: Paradermal sections of an opened *Z. mays* stoma corresponding to the middle of the GCs (**H**) and near the substomatal cavity (**I**), as seen in DIC optics. The width of the stomatal pore at these two planes is shown. Scale bars: 5 μm

During stomatal pore opening, the width of the bulbous ends increases by about 130% (Fig. 2F i), from 4.49 ± 0.03 μm to 10.36 ± 0.06 μm (n = 100) (Fig. 2B; cf. Figure 2A and Fig. 3E–G; cf. Figure 3A–D). This results at an increase in the fraction of the bulbous ends width to the central canal width to 2.16 ± 0.02 (Fig. 2F II). Furthermore, in the open stomata, the external periclinal cell wall of the bulbous ends lies on the same level with that of the central canal (Suppl. Fig. 1B). In the open stomata, the central canal swells (Fig. 2D; cf. Figure 2C) and its width increases from 3.43 ± 0.04 to 4.77 ± 0.08 μm (n = 100). During stomatal pore opening, the diameter of the central canal increases by almost 40% (Fig. 2F i). It interesting that during stomatal pore opening, the cell wall thickenings of the central canal become thinner, leading to GC cell walls that tend to be evenly thickened at the central canal region (Fig. 2D; cf. Figure 2C; also Fig. 1BIIb; cf. Figure 1BIIa). Furthermore, in open stomata, the angle θ^ο^ in Fig. 2E is reduced from 180° to 102° (n = 100; Fig. 2F iii; Fig. 3E–G; cf. Figure 3A–D), while the angle ω° in Fig. 2E is increased from 162° to 181° (Fig. 2F iii; Fig. 3E–G; cf. Figure 3A–D). The above data indicate that during stomatal pore opening, the bulbous GC ends expand anisotropically, with most of the expansion taking place in the area neighboring the polar VW ends. (Fig. 2B; cf. Figure 2A; Fig. 3E–G; cf. Figure 3A–D; Suppl. Videos 1, 2, 3).

During stomatal pore opening, the central canal of each GC is displaced towards its adjacent subsidiary cell. At the same time, the subsidiary cells change their shape and their dimensions (Fig. 2B; cf. with Fig. 2A) in order to conveniently accept the laterally displaced GC canals. According to measurements acquired from 100 closed and 100 open stomatal complexes, the width of the subsidiary cells is reduced from a mean value of 12.16 ± 0.18 μm (observed in closed stomata) to one of 6.23 ± 0.14 μm (observed in open stomata), a reduction of approximately 50%. This results in a change in the fraction of the subsidiary cells width to the whole width of the stomatal complex from 0.39 ± 0.003 to 0.23 ± 0.004 (Fig. 2F iv), demonstrating the decrease occurring in the width of the subsidiary cell during stomatal opening, as seen in median paradermal section. Simultaneously, the subsidiary cells, as seen in transverse section (Fig. 2D), curve slightly at the region of the central canal, in order to accommodate the movement of the GCs. This dynamic deformation of the subsidiary cells allows the displacement of the central canal towards them. From the study of open stomata in a series of paradermal sections, it has been shown that the width of the stomatal pore increases from the median plane towards the inner one (Figure 3H, I). Therefore, the displacement of the central canal towards the subsidiary cell is more intense at the inner side of the stomatal complex facing the mesophyll.

All the differences observed in the dimensions of the CGs and the subsidiary cells between closed and open stomatal complexes were shown to be statistically significant at the 0.001 level of significance (p ≤ 0.001). Statistical significance was assessed in SigmaPlot by Mann–Whitney Rank Sum Test.

### Distribution of cell wall matrix materials in open and closed stomata

#### Homogalacturonans: LM 20-HG epitope

**Fig. 4:**
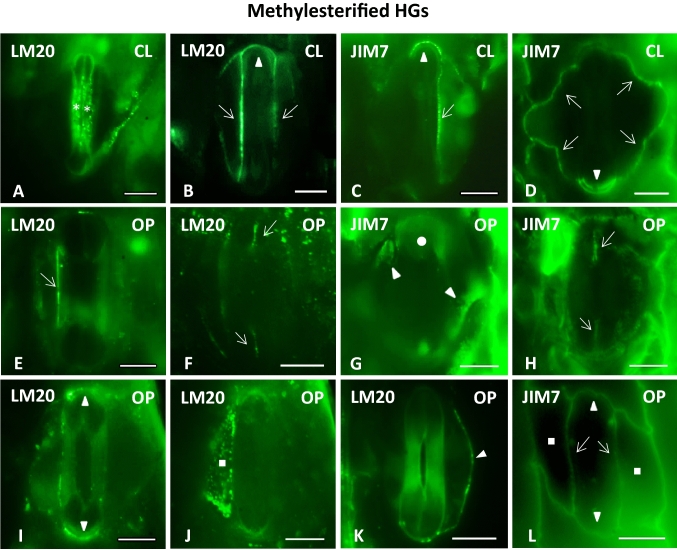
Immunolabeling of highly methylesterified HG epitopes LM20-HG (**A**, **B**, **E**, **F**, **I**, **J**, **K**) and JIM7-HG (**C**, **D**, **G**, **H**, **L**) in paradermal sections of closed (**A**–**D**) and open (**E**–**L**) stomatal complexes. *CL* closed, *OP* open. Intense fluorescent signal is emitted by the periclinal cell walls of the central canal (asterisks in **A**), the lateral cell walls of the GCs (arrows in **B**, **C**, **E**, **L**), the transverse cell walls of the GCs (arrowheads in **B**, **C**, **I**, **L**) the polar VW ends (arrows in **F**, **H**), the periclinal cell walls of the subsidiary cells (squares in **G**, **J**) and the anticlinal cell walls of the subsidiary cells (arrows in **D** and arrowhead in **K**). Note that LM20-HG deposition is observed in the periclinal cell walls of the central canal of closed stomata (**A**), while the periclinal cell walls of the subsidiary cells are enriched with both LM20-HG (**J**) and JIM7-HG (**G**) epitopes in open stomata. Scale bars: 10 μm

In closed stomatal complexes, the periclinal cell walls of the central canal appeared to be particularly enriched by the pectin epitope LM20 (Fig. 4A, 6A). This epitope, which is indicative of highly methylesterified HGs, was also detected in the lateral and transverse GC walls (Fig. 4B), while weaker fluorescence was additionally traced along the anticlinal walls of their adjacent subsidiary cells (Fig. 4B). In open stomata, the LM20-HG epitope persisted in the previously mentioned anticlinal cell wall locations (Fig. 4E, I, K) and additionally impregnated the polar VW ends (Fig. 4F). In contrast to what was observed in closed stomata (Figs. 4A, 6A), the periclinal walls of the central canals of open stomata did not emit strong LM20 fluorescent signal (Fig. 4I; cf. Figure 4A and Fig. 6A). Instead, the LM20 epitope was strongly enriching the whole extent of the periclinal cell wall of their subsidiary cells (Figs. 4J, 6A). In quantitative terms, the intensity of fluorescence, expressed as corrected integrated density per μm^2^, in the periclinal cell walls of the central canal, varied from 7442 ± 734 afu (arbitrary fluorescence units)/μm^2^ (n = 5, mean ± SE) in closed stomata to 821 ± 64 afu/μm^2^ (n = 4) in open stomata, with only the first of these measurements describing fluorescence correlated with LM20-HG epitope-specific antibody binding (Fig. 6E). In the periclinal cell walls of the subsidiary cells, the measurement of 724 ± 43 afu/μm^2^ (n = 3) made in closed stomata is once again not indicative of immunofluorescence, while the 6853 ± 538 afu/μm^2^ (n = 3) measured in the case of open stomata (Fig. 6F) strongly attests to the presence of LM20-specific fluorescent signal.

#### Homogalacturonans: JIM7-HG epitope

Though its specificity is broader than that of LM20, the JIM7 antibody is widely recognized as a molecular probe against HGs displaying a higher degree of methylesterification. On that account, similarities between the pattern of distribution of JIM7-HG and LM20-HG epitopes were expected. In both the closed and open stomatal complexes, the JIM7-HG epitope marked the lateral GC walls (Fig. 4C, L), the transverse walls of the bulbous ends (Fig. 4C, D, L) and the anticlinal cell walls of their neighboring subsidiary cells (Fig. 4D, L). On the other hand, while the periclinal cell walls of closed stomatal complexes appeared to lack fluorescent signal (Figs. 4C, D, 6B), in open stomata, a notable signal was emitted not only by the periclinal cell walls of the subsidiary cells (Fig. 6B), but also by the periclinal walls of the swollen bulbous ends (Fig. 4G). Corrected integrated density in the periclinal cell walls of the subsidiary cells of open stomata was measured at 4510 ± 719 afu/μm^2^ (n = 4) (Fig. 6F) thus further affirming JIM7-HG epitope specific antibody binding in the area. Conversely, corrected integrated density per μm^2^ in the periclinal cell wall of the subsidiary cells of closed stomata as well as in those of the central canal in both closed and open stomata was determined at 190 ± 52 afu/μm^2^ (n = 3), 113 ± 33 afu/μm^2^ (n = 3) and 350 ± 52 afu/μm^2^ (n = 4) respectively (Fig. 6E, F) and was therefore not considered indicative of JIM7-dependent immunofluorescence. In open stomata, a fluorescent signal was furthermore emitted by the polar VW ends (Fig. 4H).

#### Homogalacturonans: JIM5-HG epitope

**Fig. 5:**
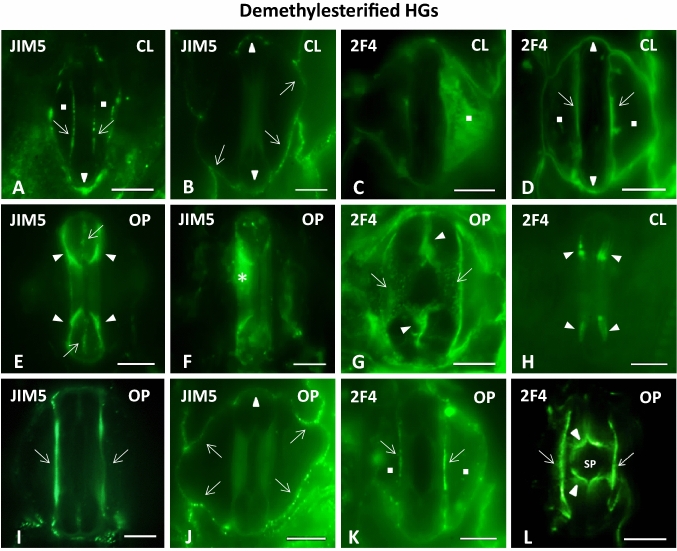
Immunolabeling of demethylesterified HG epitopes JIM5-HG (**A**, **B**, **E**, **F**, **I**, **J**) and 2F4-HG (**C**, **D**, **G**, **H**, **K**, **L**) in paradermal sections of closed (**A**–**D**, **H**) and open (**E**–**G**, **I**–**L**) stomatal complexes. *CL* closed, *OP* open. Intense fluorescent signal is emitted by the periclinal (asterisks in **F** and arrows in **G**) and the anticlinal (arrows in **A**, **D**, **I**, **K**) cell walls of the central canal, the terminal cell wall thickenings of the central canal (arrowheads in **E**, **H**), the transverse cell walls of the GCs (arrowheads in **B**, **D**, **J**), parts of the VW (arrowheads in **G**, **L**) and the periclinal (squares in **C**) and the anticlinal cell walls (arrows in **B**, **J**) of the subsidiary cells. Note that strong 2F4-HG immunofluorescence is exhibited by the periclinal cell walls of the subsidiary cells of closed stomata (**C**), while in open stomata the periclinal cell walls of the central canal are enriched in JIM5-HG (**F**) and 2F4-HG (**G**) alike. *SP* Stomatal Pore. Scale bars: 10 μm

**Fig. 6:**
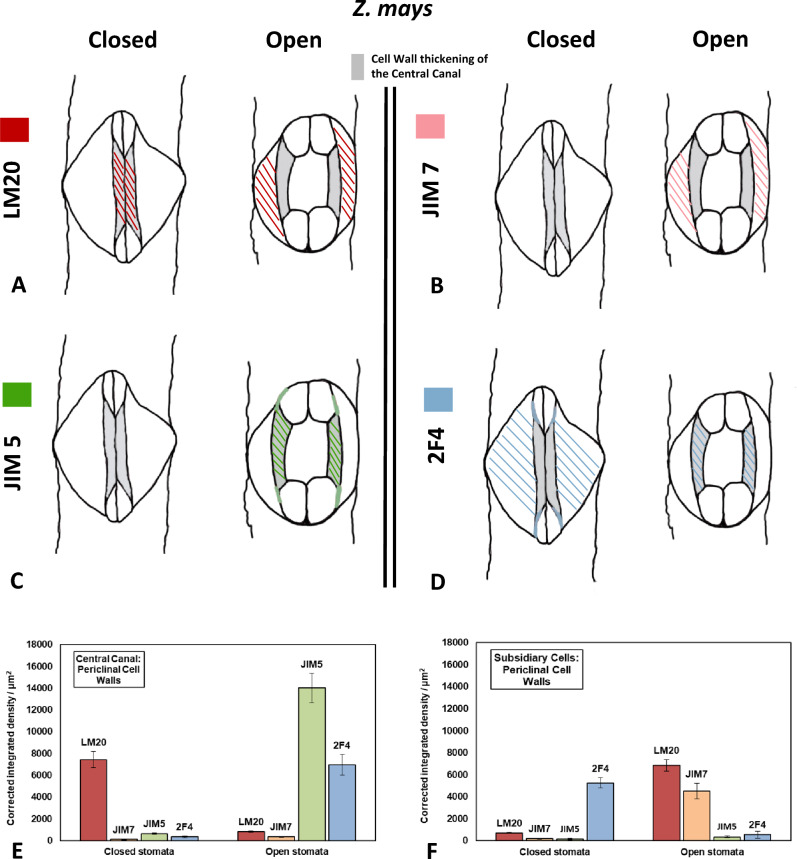
HG epitopes in the periclinal cell walls of the central canal and of the subsidiary cells in closed and open stomata of *Z. mays*. **A**–**D**: Diagrammatic representation of the distribution of pectin epitopes in the periclinal cell walls of the central canal and of the subsidiary cells in closed and open stomata. LM20 (**A**) is shown in red, JIM7 (**B**) in orange, JIM5 (**C**) in green and 2F4 (**D**) in blue. The cell wall thickenings of the central canal appear in gray. Signal detected at the terminal thickenings of the central canal is also depicted (**C, D**). **E**, **F**: Quantification of fluorescence expressed as corrected integrated density/μm^2^ in the periclinal cell walls of the central canal (**E**) and of the subsidiary cells (**F**) in closed and open stomata after incubation with antibodies LM20 (red), JIM7 (orange), JIM5 (green) and 2F4 (blue). Corrected integrated density/μm^2^ is measured in arbitrary fluorescence units (afu)/μm^2^ set by ImageJ

The JIM5-HG epitope contains few methyl esters and as a rule identifies HGs presenting a considerable degree of demethylesterification. In closed stomata, the epitope was preferentially deposited across the lateral walls of the central canal (Fig. 5A), the transverse walls of the bulbous ends (Fig. 5A, B) and the anticlinal cell walls of the subsidiary cells (Fig. 5B). At the same time, it was distinctively absent from the periclinal cell walls of the stomatal complex (Fig. 6C). Indeed, in the periclinal cell walls of both the central canal and the subsidiary cells of closed stomata, corrected integrated density per μm^2^ was particularly low, having been measured at 636 ± 78 afu/μm^2^ (n = 4) and 160 ± 78 afu/μm^2^ (n = 4) (Fig. 6E, F). In contrast, in open stomatal complexes, a JIM5 fluorescent signal was prominent in the periclinal wall of the central canal (Figs. 5F, 6C), including, most notably, its terminal thickenings (Fig. 5E). Corrected integrated density on the central canal of open stomata was specifically measured at 14,027 ± 1328 afu/μm^2^ (n = 3) (Fig. 6E), heavily contrasting the 358 ± 98 afu/μm^2^ (n = 5) measured in the periclinal cell walls of the subsidiary cells of open stomata (Fig. 6F), where no JIM5-dependent fluorescent signal was detected (Fig. 6C). All the while, the JIM5-HG epitope continued impregnating the lateral (Fig. 5I) and transverse (Fig. 5J) GC walls as well as the anticlinal subsidiary cell walls (Fig. 5J). A weaker JIM5 signal was additionally traced in the polar VW ends (Fig. 5E).

#### Homogalacturonans:2F4-HG epitope

The 2F4 monoclonal antibody recognizes demethylesterified pectins that are crosslinked by way of calcium ion bridges. In both closed and open *Z. mays* stomata, the 2F4-HG epitope was present in the lateral GC walls (Fig. 5D, K, L), the transverse cell walls of the bulbous ends (Fig. 5D, K) and the anticlinal walls of the subsidiary cells (Fig. 5D, G). In contrast, only in open stomata was a clear 2F4 fluorescent signal noted in the VWs, indicating the deposition of this specific subcategory of HGs around the opened stomatal pore (Fig. 5L), as well as on the surface of the polar VW ends (Fig. 5G). In the periclinal walls of stomatal complexes a notable shift was observed in the distribution of the 2F4-HG epitope, which contrasted that described for the LM20-HG epitope. Specifically, while the closed stomatal complexes exhibited strong fluorescence in the periclinal walls of their subsidiary cells (Figs. 5C, 6D), these same walls did not emit signal in open stomata (Fig. 6D). In their case, the 2F4-HG epitope was present in the periclinal walls of the central canal instead (Figs. 5G, 6D). In quantitative terms this shift was illustrated in the periclinal cell walls of the subsidiary cell by a transition from a corrected integrated density of 5273 ± 445 afu/μm^2^ (n = 8) in closed stomata to one of only 558 ± 316 afu/μm^2^ (n = 6) in open stomata (Fig. 6F). Conversely, corrected integrated density at the periclinal cell walls of the central canals rose from a low measurement of 350 ± 106 afu/μm^2^ (n = 6) in closed stomata to one of 6986 ± 954 afu/μm^2^ in open stomata (n = 6) (Fig. 6E). 2F4-dependent fluorescence was lastly traced in the terminal thickening of the central canal (Fig. 5H), as it is the case with JIM5-HG epitope (Fig. 5E).

#### Xyloglucans: LM15 epitope

**Fig. 7:**
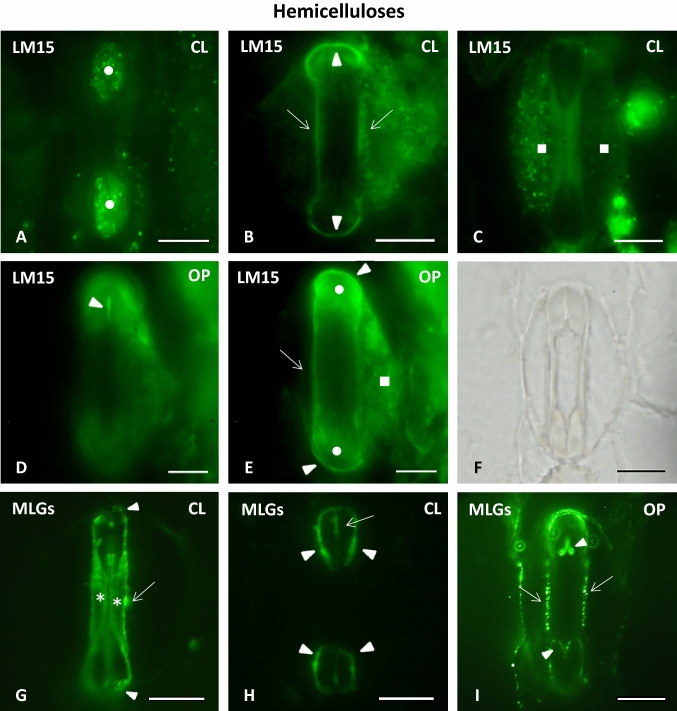
Immunolabeling of xyloglucans (LM15 epitope) (**A**–**E**) and MLGs (BG-I) in paradermal sections of closed (**A**–**C**, **G**, **H**) and open (**D**, **E**, **I**) stomatal complexes. *CL* closed, *OP* open. Intense LM15 fluorescent signal is emitted by the periclinal cell walls of the bulbous ends (circles in **A**, **E**), the polar VW ends (arrowheads in **D** and arrows in **H**), the lateral GC cell walls (arrows in **B**, **E**), the transverse GC cell walls (arrowheads in **B**, **E**) and the periclinal cell walls of the subsidiary cells (squares in **C**, **E**). BG-1 fluorescent signal is exhibited by the anticlinal (arrow in **G**), the lateral (arrow in **I**) and the transverse GC walls (arrowheads in **G**, signal also visible in **H**, **I**), the periclinal cell walls of the central canal (asterisks in **G**), the terminal cell wall thickenings of the central canal (arrowheads in **H**) and parts of the VW (arrows in **H** and arrowheads in **I**). **F**: the stomatal complex of I as seen in DIC optics. Scale bars: 10 μm

In closed stomata, this prominent group of hemicelluloses strongly enriched the periclinal walls of the bulbous ends (Fig. 7A), while the LM15 epitope additionally marked the whole length of both the lateral and the transverse GC walls (Fig. 7B). At the same time, LM15 fluorescent signal was also emitted by the subsidiary cell walls of closed stomatal complexes, whose periclinal walls appeared strongly impregnated with the epitope (Fig. 7C). In open stomata, the LM15 fluorescent signal persisted along the lateral and the transverse GC walls as well as in the periclinal walls of both the bulbous ends and the subsidiary cells (Fig. 7E). However, in open stomata, a deposition of xyloglucans could also be observed in the polar VW ends (Fig. 7D), where no fluorescence was detected in the closed stomata.

#### Mixed Linkage Glucans: BG-1 epitope

The immunolocalization of MLGs revealed a remarkably broad distribution of their epitope in *Z. mays* stomatal complexes. In closed stomata, MLGs deposition was practically detected in the entirety of GC cell walls. Intense fluorescence was emitted by the periclinal cell walls of the central canal (Fig. 7G), including those of its terminal thickenings (Fig. 7G, H). MLGs were, moreover, found in the lateral GC cell walls (Fig. 7G), while a fluorescent signal was also emitted by the polar VW ends (Fig. 7H) and the transverse GC cell walls (Fig. 7G, H). In open stomata, MLGs visibly impregnated the lateral and the transverse GC cell walls (Fig. 7I), whereas a fluorescent signal was obvious at the parts of the VW that line the opposite ends of the stomatal pore (arrowheads in Fig. 7I).

## Discussion

### General remarks on dumbbell-shaped stomatal movement

This work describes for the first time in detail the major structural modifications and geometrical changes that occur during the opening and the closure of *Z. mays* stomatal complexes*.* Turgor pressure increase in GCs (review by Nunes et al. 2020), triggers the following highly controlled structural changes leading to the stomatal pore opening:

1. The bulbous GC ends bulge anisotropically (Suppl. Video 3; cf. Suppl. Video 2). The terminal cell wall thickenings that extend up to the junction site of the lateral cell wall of the GC with the periclinal cell wall of the bulbous GC ends do not allow any deformation to occur at this site. On the contrary, the part of the VW between the central canal and the polar VW end is extended (see arrowheads in Fig. 2B). This results in the volume increase of the bulbous ends towards the polar ends of the VW. No detectable changes occurred at the contact site of the bulbous ends with the neighboring subsidiary cells.
2. The central canals increase in diameter, and at the same time are laterally displaced towards the adjacent subsidiary cells, leading to the opening of the stomatal pore. The swelling central canals display a change in the thickness of their cell wall thickenings. The mechanical tension causing their lateral displacement is applied by the anisotropically bulged bulbous GC ends.
3. During displacement, the central canals appear to be rotating slightly towards the subsidiary cells. Such a rotation could result in a larger opening of the stomatal pore on the side of the mesophyll. Simultaneously, the volume of the subsidiary cells decreases, and their shape changes adopting a characteristic curvature due to the displacement of the central canals of the GCs. This change is biomechanically important, since it allows the lateral displacement of the central canal of the neighboring GC “inside” the space that was previously occupied by the subsidiary cell (see also Franks and Farquhar 2007; Giannoutsou et al. 2020).

The closure of the stomatal pore is accompanied by the following structural changes:

1. After decrease of turgor, the bulbous ends of the GCs cease to exert forces on GC central canals, while simultaneously the subsidiary cells expand due to the water influx from the GCs and exert mechanical stress mainly on the central canals, forcing them to approach each other. The stomatal pore thus closes.
2. During stomatal pore closure, the diameter of the central canals decreases, while the periclinal cell wall appears more intensely thickened.
3. Simultaneously with the reduction of the volume of the central canal, the bulbous GC ends shrink and their periclinal cell walls are elevated to a higher plane than those of the central canal. It is possible that, during this course, the shrinking central canal exerts mechanical tensions on the bulbous GC ends. Following the occurrence of all the phenomena, the GCs acquire their original dumbbell shape.

### GC bulbous ends deformation

**Fig. 8:**
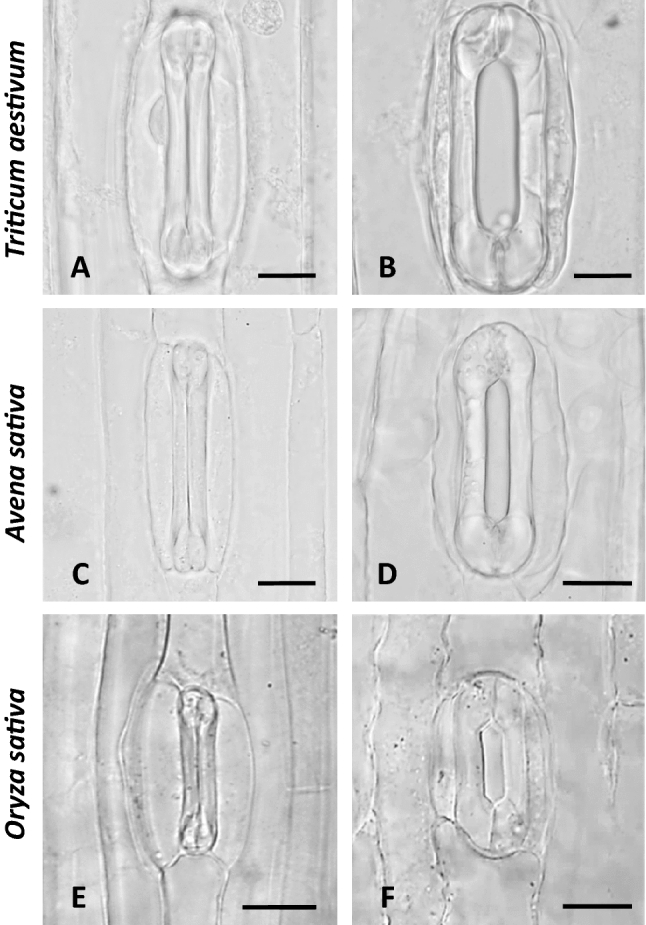
Closed (**A**, **C**, **E**) and open (**B**, **D**, **F**) stomatal complexes of three grasses species as seen in paradermal sections observed by DIC optics. Scale bars: 10 μm

The observations and measurements made in this work show clearly that the anisotropical expansion and asymmetric deformation of the bulbous ends is a common feature of the dumbbell-shaped stomatal complexes of grasses. Apart from *Z. mays*, it has also been observed in the *Triticum aestivum*, *Avena sativa* and *Oryza sativa* (Fig. 8). The cellulose microfibril systems of the periclinal walls that radiate out from the ends of the central canal (Ziegenspeck 1939, 1944; Galatis 1980) allow the tangential expansion of the periclinal cell walls of the bulbous ends and their bulging, when the turgor pressure increases. In that way, they could behave similarly to those in the periclinal cell walls of the kidney-shaped GCs (Galatis and Apostolakos 2004; Marom et al. 2017; Woolfenden et al. 2017; Bidhendi and Geitmann 2018).

The terminal cell wall thickenings of the central canal are rich in cellulose microfibrils arranged almost parallel to the GC axis (Galatis 1980; Shtein et al. 2017; see also Fig. 1F), display high stiffness (Shtein et al. 2017; Rui et al. 2018) and are impregnated with callose (Giannoutsou et al. 2020; Apostolakos et al. 2021). These features prevent the expansion of the bulbous ends towards the subsidiary cells and force them to ‘‘curve’’, to some extent, inwards. Thus, they may provide a reasonable explanation for the anisotropical swelling of the bulbous ends. The deformed bulbous ends exert intense mechanical stress on the terminal thickenings of the central canal and the polar VW ends. The latter are rich in crystalline cellulose (Shtein et al. 2017; see also Suppl. Fig. S2A) and callose (Giannoutsou et al. 2020; Apostolakos et al. 2021; see also Suppl. Fig. S2C) and can therefore withstand the applied mechanical stress and transfer it to the central canals.

The periclinal cell walls of the bulbous ends as well as their transverse cell walls are rich in xyloglucans (LM15 epitope; see Fig. 7A, E) and in methylesterified HG epitopes (detected by LM20 and JIM7 antibodies; see Fig. 4G). These matrix materials favor elasticity and promote the expansion of the cell wall, facilitating the increase in the volume of the bulbous ends. Another factor facilitating the swelling of the bulbous ends is the absence of crystalline cellulose from their periclinal cell walls (Shtein et al. 2017), which allows for the mutual cellulose microfibril distancing. Finally, during the asymmetrical expansion of the bulbous ends, the VW regions of the bulbous ends that line the edges of stomatal pore elongate (arrowheads in Fig. 2B; compare to Fig. 2A). In closed stomata these cell wall regions, which are far less thickened (arrowheads in Fig. 1C) than the terminal thickenings of the central canal, have been shown to lack crystalline cellulose (Shtein et al. 2017), in a way that favors their expansion during the opening of the stomata. In open stomata, however, these same parts of the VW which extend between the central canal and the polar VW ends, appear to not only contain crystalline cellulose (Suppl. Fig. 2B) but also callose (Suppl. Fig. 2D) and the 2F4-HG epitope (Fig. 5L).

The deposition of 2F4-HG epitope in the cell wall is not necessarily incompatible with its ability to stretch. Even in regions dominated by demethylesterified HGs linked together by calcium bridges, homogalacturonan-degrading enzymes can be activated and set in motion pectin network recycling and matrix reorganization, thereby inducing cell wall relaxation and possibly allowing its expansion (Willats et al. 2001; Verhertbruggen and Knox 2007; Pelletier et al. 2010; Wolf and Greiner 2012; Bidhendi and Geitmann 2016; Giannoutsou et al. 2016). On the other hand, however, it is not excluded that 2F4-HG epitope plays the expected role at the parts of the VW located between the central canal and the polar ends of the VW (Fig. 5L), as well as in the polar VW ends (Fig. 5G) and in the terminal thickenings of the central canal (Fig. 5H). The noted presence of crystalline cellulose and callose in the parts of the VW between the central canal and the polar VW ends (Suppl. Fig. 2B, D) could therefore also be involved in further stabilizing these cell wall regions in open stomata.

After all, achieving the maximum stomatal aperture is not always the desired goal. In contrast, stomatal aperture, especially in a C_4_ grass such as *Z. mays*, is carefully adjusted at the size needed to successfully perform gas exchange in the given environmental conditions. The presence of Ca^2+^-binding HGs, callose and crystalline cellulose may precisely serve this purpose: to maintain stomatal aperture at an ideal level (see Fig. 2B, Fig. 3).

### Central canal deformation

The observation that the diameter of the central canal of maize stomata undergoes a slight increase during stomatal opening and a respective decrease during stomatal closure mirrors the results of Nunes et al. (2022) who report that the diameter of the central canal in the notably smaller stomata of *Brachypodium distachyon* varied from 2.48 ± 0.3 μm when the stomatal pore was closed to 3.52 ± 0.6 μm when the stomatal pore was open. Since, within existing literature, vacuoles have only been observed in the bulbous ends of dumbbell shaped stomata (Galatis 1980; Sack 1987 and references therein), the exact way in which this reversible change in the width of the central canal takes place remains unclear. Nevertheless, the present study is the first to report that the augmentation of the central canal’s diameter during stomatal pore opening is accompanied by a reduction of its cell wall thickenings, which are then reinstated at their original condition during stomatal closure, in parallel with the shrinkage of the central canal. The fine tuning of the reorganization of the cell wall materials is necessary for these changes to occur.

Cellulose microfibril reorganization has been reported during kidney-shaped stomatal function in *Arabidopsis thaliana*, where cellulose microfibrils of the periclinal cell walls of the GCs display a relatively uniform distribution in the open stomata and a more fibrillar pattern in the closed ones (Rui and Anderson 2016). In this reorganization of cellulose microfibrils hemicelluloses and pectins seem to be involved (Rui and Anderson 2016; Rui et al. 2017, 2018). Cellulose microfibrils at the cell wall thickenings of the central canal of the dumbbell-shaped GCs tend to be arranged along the stomatal axis (Ziegenspeck 1939, 1944; Galatis and Apostolakos 2004). In these areas, cellulose does not display a high degree of crystallinity (Shtein et al. 2017). At the central canal region, crystalline cellulose is mainly located at the VW sites lining the median region of the stomatal pore (see Figs in Shtein et al. 2017).

The periclinal cell wall thickenings of the central canal in closed *Z mays* stomata are rich in LM20- HG epitope (Fig. 6A, E). The strong methylesterification of HGs is associated with the formation of low-viscosity gels that facilitate cell wall relaxation, making it more extensible and deformable (Verhertbruggen and Knox 2007; Wolf and Greiner 2012; Bidhendi and Geitmann 2016). These pectin epitopes possibly make the cell wall thickenings of the central canal capable of expansion. Their deposition may therefore prepare the central canal for the forthcoming stomatal opening, during which the central canal is subject to a certain swelling and the periclinal cell wall needs to possess a certain level of elasticity.

It is interesting that these methylesterified HGs, which presumably moderate the rigidifying effect of crystalline cellulose, were not detected at the periclinal cell walls of the central canal in open stomata (Fig. 6A, E). There, they were replaced by epitopes of demethylesterified HGs (JIM5-HG, 2F4-HG epitopes; see Fig. 6C–E), which are correlated with the local stiffening of the cell wall matrix, thus increasing the overall stability of the cell wall (Verhertbruggen and Knox 2007). In other words, the assumed relative extensibility of the periclinal cell walls of the central canal in closed stomata is transitory and is replaced in open stomata by stiffness. Their capacity for rapid recycling frames HGs as polymers better suited for the readjustment of the mechanical properties of GC walls that inevitably occur during the reversible stomatal movements, in comparison to less flexible molecules like xyloglucans (see Samaj et al. 2005).

Nevertheless, it is probably in combination with hemicelluloses (MLGs; see Fig. 7G), that HGs contribute to the mutual distancing and rapprochement of the cellulose microfibrils of the central canals during *Z. mays* stomatal function, similarly to what happens in kidney-shaped stomata (Rui and Anderson 2016; Rui et al. 2017, 2018). The contribution of hemicelluloses and pectins to the functional mechanism of *Z. mays* stomata has also been supported by Jones et al. (2005). The low degree of cellulose crystallinity in the region of the central canal (Shtein et al. 2017) may positively affect the mutual distancing of the cellulose microfibrils as well. Finally, the callose located at the cell wall thickenings of *Z. mays* central canal (Giannoutsou et al. 2020) contributes to the stability of this region during the opening and closure of the stomatal pore (Αpostolakos et al. 2021).

The lateral displacement of the central canals towards the subsidiary cells during the opening of the stomatal pore was initially described by Franks and Farquhar (2007) and has been confirmed by Giannoutsou et al. (2020) and the data of the present work. As it has already been mentioned, the mechanical forces driving the displacement should be applied by the anisotropically expanded GCs bulbous ends. Apostolakos et al. (2021) have supported that the terminal cell wall thickenings of the central canal, by “acting as levers”, apply this mechanical tension at the central canal causing its displacement. Our data show that, as the central canal is displaced, it simultaneously rotates, to some extent, towards the subsidiary cell, leading to an increased stomatal pore opening at the side of the mesophyll (see Fig. 3H, I). This rotation becomes possible because, during the formation of the substomatal cavity, partial detachment of the lateral anticlinal cell walls takes place (Galatis 1980). It subsequently results in a higher degree of movement of the central canal towards the subsidiary cell. From the figures published in Srivastava and Singh (1972) and Galatis (1980) it can be concluded that the terminal cell wall thickenings of the central canal are stronger at the internal GC site than at the external one (see asterisks in Fig. 1D). This structural difference possibly allows the application of a stronger mechanical stress at the inner sites of the central canals forcing their displacement and their simultaneous rotation towards the subsidiary cell.

As stated in the work of Apostolakos et al. (2021), the central canal of dumbbell-shaped stomata and its terminal thickenings function as a united system. It should therefore be expected that mechanical forces generated during stomatal closure by the reinstatement of the cell wall thickenings in the main body of the central canal would be transmitted to its terminal thickenings. Such mechanical tension could possibly force the base of the terminal thickenings of the central canal inwards, thus complementing the shrinkage of the bulbous ends and establishing, in the process, the slight outwards tilt displayed by the lateral cell walls of the bulbous ends in closed stomata (see angle ω^o^ in Fig. 2). The combined forces applied by the terminal thickenings of the central canal and the thickened and tightened central canal itself would probably not leave the relatively thinner periclinal cell walls of the bulbous ends unaffected either. Facilitated by both their enrichment in xyloglucans (see Fig. 7A, E) and their systems of radially arranged cellulose microfibrils, the periclinal cell walls of the bulbous ends could curve, to a degree, upwards, thus explaining their observed elevation in relation to those of the central canals of closed stomata.

Recent findings have supported our data on the importance of the cell wall thickenings of the GC central canal on grass stomatal function. Durney et al. (2023) have created a finite element method model of grass stomatal complexes to shed light into the mechanics of stomatal opening and closure. Between their observations, they pinpoint the importance of a relatively thick GC central canal region for the enhancement of pore opening. Furthermore, Zhou et al. (2023) identified a maize gene encoding UDP-glucose 4-epimerase that regulates the supply of UDP-glucose during GC wall synthesis. The lack of gene function leads to the reduction of cellulose and MLGs synthesis in the GCs, resulting in impaired local cell wall thickening. In the mutants examined, the central canals had collapsed, and the local cell wall thickenings were absent, leaving the remains of the two bulbous ends. The authors suggest that BZU3 is specifically required at the later stage of stomatal elongation, when the GCs become locally thickened at the central canal. They also attribute the highly specialized shape and consequent function of grass stomata to the spatiotemporal activity of specific cell wall enzymes at highly important cell wall sites.

### Subsidiary cells deformation

During the function of dumbbell-shaped stomata, significant changes occur in the shape and the size of the subsidiary cells. When compared with that in closed stomatal complexes, the width of the subsidiary cells in open stomata is decreased by almost 50%. These structural changes probably require fine rearrangements of the cell wall materials. The microtubules that line the periclinal cell walls of the mature subsidiary cells of *Z. mays* appear randomly distributed (Panteris et al. 2007). The respective deposition of the cellulose microfibrils allows the expansion of the periclinal cell walls when mechanical stress is exerted to them during the inward displacement of the GC central canal when the stomatal pore opens. Furthermore, a combination of preexisting data (Giannoutsou et al. 2020) with those presented here show that the cell walls of the subsidiary cells are rich in methylesterified HGs and xyloglucans (LM20, JIM7 and LM15 epitopes; see Fig. 6A, B, F and Fig. 7C, E respectively). By favoring cell wall elasticity (Woolfenden et al. 2017; Shtein et al. 2017), these matrix materials, permit the deformation of the cell wall during stomatal pore opening and closure. It is interesting that during stomatal pore movement, intense 2F4-HG signal is detected at the cell walls shared with the subsidiary cell and the central canal (Fig. 5D, K, L), as in this region, strong mechanical forces are exerted on the cell wall by the bulging subsidiary cell.

Although the anticlinal walls of the subsidiary cells receive much of the pressure that develops during central canal displacement, their periclinal walls do not remain unaffected either. Although this fact can be discerned in the cryo-electron microscopy images of Franks and Farquhar (2007), it is even more pronounced in cross-sections of *Z. mays* stomatal complexes (Giannoutsou et al. 2020; see also Fig. 2D), in which the periclinal cell walls of the subsidiary cells appear to deform, increasing their curvature and elevating their central region. Such a change in the curvature of the periclinal walls of the subsidiary cells justifies their enrichment with the LM20-HG epitope in the open stomatal complexes (Fig. 6A, F).

In contrast to what has been observed in open stomata, the periclinal cell walls of the subsidiary cells of closed stomata emitted a strong 2F4-HG fluorescent signal (Fig. 6D, F). While the presence of this epitope does not rule out cell wall malleability, it is well documented that the deposition of Ca^2+^-linked HGs in their periclinal walls can possibly reinforce the capacity of the subsidiary cells to exert the mechanical forces that are necessary for pushing the central canals inwards and closing the stomatal pore. It is moreover interesting that the shifts described regarding the HG content of the periclinal cell wall of the subsidiary cells are almost opposite to those observed in the periclinal cell walls of the central canal (Fig. 6), thus implying that HGs could be crucial participants in the “see-sawing” of mechanical forces between the central canal and the subsidiary cells during the reversible movements of dumbbell-shaped stomata.

What should hereby be noted is that the previously mentioned shifts are not only keeping pace with changes in the shape of subsidiary cells and guard cells but also with major fluctuations of their surface and volume. While to our knowledge such measurements have not yet been performed on dumbbell-shaped stomata, kidney shaped GCs have been shown to undergo a 30–40% increase in their surface area and a twofold to threefold increase in their volume during stomatal opening (Larson et al. 2017 and references therein). As noted by Meckel et al. (2007), these changes, which are then reversed as the stomata close, cannot be accommodated by the limited elasticity of biological membranes, requiring thus the recycling of membranes through endocytosis and exocytosis. Indeed, both endosomal phosphatidylinositol 3-phosphate (Park et al. 2003) and clathrin heavy chain subunits (Larson et al. 2017) have been established as essential for normal stomatal function. What is then particularly intriguing, is the fact that in growing and dividing cells measurable amounts of cell wall material, including, most notably, cross-linked pectins, are internalized in membranous endosomal compartments (Šamaj et al. 2005 and references therein). As proposed by Šamaj et al. (2005) a similar process could be taking place in stomatal complexes, where cell wall pectins could be internalized and then recycled back to the cell wall, with or without modifications such as the breakage of their cross-linkages. This suggestion is compatible with the shifts in the distribution of HGs that are observed in this work, even more so when taking into account that stomatal opening or closure in grasses can occur in a timespan as short as three minutes (Nunes et al. 2022).

Previous work on stomatal ontogenesis in grasses revealed that orthologues of the *Arabidopsis* stomatal bHLH transcription factor MUTE are essential for the establishment of subsidiary mother cell’s identity in the three grass species *Brachypodium distachyon*, *Zea mays* and *Oryza sativa* and that the *mute* mutants lack subsidiary cells (Raissig et al. 2017; Wang et al. 2019; Wu et al. 2019; Zhang et al. 2022). In a *B. distachyon mute* mutant, the lack of the subsidiary cell formation results in smaller maximal pore area and lower gas exchange capacity (Raissig et al. 2017). These plants produced, after 5-week growth, less biomass. These results support that subsidiary cells formation is crucial for stomatal function and rapid response to environmental changes (Zhang et al. 2022).

Recently, Durney et al. (2023) reported that, according to their estimation, although subsidiary cells are not required for stomatal function, they can enhance stomatal closure. At another study, Liu et al. (2023) compared stomatal gas exchange and stomatal aperture dynamics in various *Z. mays* mutants that display varying percentages of aberrantly formed subsidiary cells. They confirm that stomata with one or two defective subsidiary cells cannot close properly, thus highlighting that subsidiary cells are essential for proper stomatal function. Furthermore, in their results, it is worth noticing that in stomata with one abnormal subsidiary cell and one normal cell, the GCs tended to be curved away from the normal subsidiary cell. This observation strengthens the notion that the subsidiary cells apply a force on the central canal that drives stomatal closure. When the subsidiary cell is missing, the opposite force from the second subsidiary cell is not applied and this results in the curved GCs displayed (Liu et al. 2023).

## Conclusion

The present work finally confirms the observations of Aylor et al. (1973), demonstrating for the first time in quantitative terms that the swelling of the bulbous ends of dumbbell-shaped GCs does not occur symmetrically but asymmetrically, taking place mainly in the region near the VW. Such an asymmetry is compatible with the limitations placed on the extension of the bulbous cell walls by the radial arrangement of cellulose microfibrils in the periclinal cell walls and the unique characteristics of the terminal cell wall thickenings of the central canal. The differential distribution of important cell wall matrix materials across the cell walls of *Z. mays* stomatal complexes, together with the proposed rearrangements that it undergoes during stomatal opening and closing, suggest that the cell wall matrix plays an important role in facilitating the reversible movements of the dumbbell-shaped grass stomata.

### Supplementary Information

Below is the link to the electronic supplementary material.

Supplementary file1 (DOCX 112 KB)

Supplementary file2 (MP4 75750 KB) Suppl. Video 1. 3D reconstruction video created by CLSM images of Z. mays epidermis taken by BC-43 confocal microscope. The 3D reconstruction has been created using Imaris-10 software

Supplementary file3 (MP4 180191 KB) Suppl. Video 2. 3D reconstruction video created by CLSM images of a Z. mays open stoma taken by BC-43 confocal microscope. The 3D reconstruction has been created by the use of Imaris-10 software

Supplementary file4 (MP4 149455 KB) Suppl. Video 3. 3D reconstruction video created by CLSM images of a Z. mays closed stoma taken by BC-43 confocal microscope. The 3D reconstruction has been created by the use of Imaris-10 software

## Data Availability

The data that support the findings of this study are not openly available due to reasons of sensitivity and are available from the corresponding author upon reasonable request. Data are located in controlled access data storage at the Biology Department of the National and Kapodistrian University of Athens.

## Abbreviations

GCs: Guard cells
VW: Ventral cell wall
HG: Homogalacturonans
MLG: Mixed linkage glucans
