# Supplementary material for: Cell wall anisotropy plays a key role in Zea mays stomatal complex movement: the possible role of the cell wall matrix
Source: Plant Mol Biol. 2023 Dec 18;113(6):331–51. doi: 10.1007/s11103-023-01393-x (PMC10730690; doi:10.1007/s11103-023-01393-x)

**Suppl. Fig. 1** Optical paradermal sections of a closed (A) and an open (B) *Z. mays* stomatal complex. The optical sections are focused at the surface of the bulbous ends. In (A), the central canal is not clearly seen, while in (B), the central canal and the bulbous ends are focused on the same plane. Scale bars:10 μm

**Suppl. Fig. 2**

**A, B:** Immunolabeling of crystalline cellulose in closed (A) and open (B) *Z. mays* stoma. Crystalline cellulose is deposited at the lateral cell wall of the GCs and at the polar VW ends in the closed stoma (arrow and arrowhead in A) as well as at the VWs that line the open stomatal pore (arrowhead in B). The squares mark the subsidiary cells. SP: Stomatal Pore. Scale bars:10 μm.

**C**: Callose immunolocalization in an open *Z. mays* stomatal complex. Callose is located at the polar VW ends (arrows) as well as at the transverse cell walls of the GCs (arrowheads). The squares mark the subsidiary cells. Scale bar: 10 μm

D: Median paradermal optical section of an open *Z. mays* stoma, as seen after aniline blue staining. A fluorescent signal is emitted by the parts of the VW that line the stomatal pore (arrowheads). SP: Stomatal Pore. Scale Bar: 10 μm.


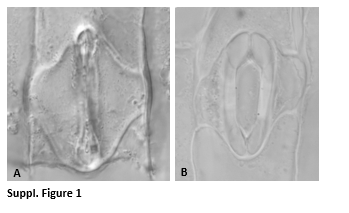


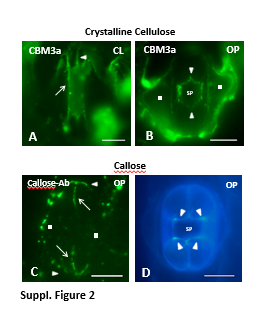

Supplement: Supplementary file 1 — Supplementary file1 (DOCX 112 KB) [file 11103_2023_1393_MOESM1_ESM.docx]
